# Prognosis of single tooth implants following alveolar ridge preservation with two recombinant human bone morphogenetic protein-2 delivery systems

**DOI:** 10.1186/s12903-021-01565-5

**Published:** 2021-04-20

**Authors:** Hyeong-Jin Baek, Il-hyung Kim, Pil-Young Yun, Young-Kyun Kim

**Affiliations:** 1 Department of Oral and Maxillofacial Surgery, Section of Dentistry, Seoul National University Bundang Hospital, 82 Gumi-ro 173beon-gil, Bundang-gu, 13620 Seongnam, Korea; 2 Office of Human Resources Development, Armed Forces Capital Hospital, Armed Forces Medical Command, Seongnam, Korea; 3 School of Dentistry and Dental Research Institute, Seoul National University, Seoul, Korea

**Keywords:** Alveolar bone loss, Alveolar process, Bone substitutes, Dental implants, rhBMP-2 protein, Survival rate

## Abstract

**Background:**

We previously reported similar efficacies of alveolar ridge preservation (ARP) on single extraction socket with two different* E. coli* derived recombinant human bone morphogenetic protein-2 (rhBMP-2) delivery systems (Cowell BMP, Cowell medi Co, Busan, Korea; β-tricalcium phosphate and hydroxyapatite particle & O-BMP, Osstem Implant Co, Busan, Korea; absorbable collagen sponge). After the trial, we completed implant therapy and observed over an average of 3 years. This follow-up study was performed retrospectively to compare result of implant treatment at the preserved alveolar ridge site.

**Methods:**

Patients who underwent extraction of single tooth and received ARP with one of two rhBMP-2 delivery systems from October 2015 to October 2016 were enrolled. Twenty-eight patients (Group 1: Cowell BMP 14; Group 2: O-BMP 14) who underwent implant therapy and prosthetic treatment were included in study. Stability and marginal bone loss (MBL) of each implant were collected from medical charts and radiographs, and analyzed. The survival and success rates of implants were calculated.

**Results:**

The primary implant stability represented by implant stability quotient (ISQ) for Groups 1 and 2 was 69.71 and 72.86, respectively. The secondary implant stability for Groups 1 and 2 was 78.86 and 81.64, respectively. Primary and secondary stabilities were not statistically different (*P* = 0.316 and 0.185, respectively). MBL at the latest follow-up was 0.014 mm in Group 1 over 33.76 ± 14.31 months and 0.021 mm in Group 2 over 40.20 ± 9.64 months, with no significant difference (*P* = 0.670). In addition, the success rate of implants was 100% (14/14) in Group 1 and 92.9% (13/14) in Group 2, with survival rate of 100% (14/14) in Group 1 and 92.9% (13/14) in Group 2.

**Conclusions:**

We confirmed good prognosis in both groups as a result of implant therapy after ARP with each of two rhBMP-2 carriers.

## Background

Maintenance and preservation of alveolar width and height are important for proper dental implant placement. However, it is difficult to place implants in areas of previous tooth extraction since alveolar bone is prone to horizontal and vertical absorption [1, 2]. Therefore, several procedures including ridge preservation have been introduced to maintain the volume and shape of alveolar bone after tooth extraction, but the effectiveness of these procedures remains controversial [2, 3]. The use of autogenous bone for alveolar augmentation and preservation has been considered the gold standard [4]. However, complications including donor site comorbidity, limited amount of harvest, resorption after grafting, and difficulty in harvesting because of anatomical and underlying diseases make difficulties to use autogenous bone [3, 4]. So, bone substitutes including allogenic, xenogenic, and alloplastic materials have been used as alternatives to autogenous bone graft, but each has limitations as well [5–9]. As a result, osteoinductive materials have been considered and used for prevention of alveolar bone resorption. Among them, recombinant human bone morphogenetic protein-2 (rhBMP-2) has been authorized to be used for alveolar ridge and maxillary sinus augmentation in the maxillofacial area [10]. Several studies have shown that bone grafting with rhBMP-2 at alveolar bone defect sites resulted in clinically and histologically similar volume and quality of alveolar bone compared to outcomes of grafting with autogenous bone [11–13]. Therefore, it is believed that rhBMP-2 is an appropriate material for post-extraction alveolar bone preservation [14]. However, it is necessary to identify proper carriers of rhBMP-2 for optimal bone regeneration through osteoinductivity since rhBMP-2 tends to be absorbed when used alone [15–17].

We previously compared the efficacy of two rhBMP-2 delivery systems (Cowell BMP, Cowell medi Co, Busan, Korea & O-BMP, Osstem Implant Co, Busan, Korea) on alveolar bone preservation. And we concluded from the 12-week clinical trial that the β-tricalcium phosphate and hydroxyapatite particle delivery system with *E. coli*-derived rhBMP-2 (Cowell BMP) and newly developed absorbable collagen sponge delivery system with *E. coli* derived rhBMP-2 (O-BMP) showed similar efficiency for the alveolar ridge preservation (ARP). [18].

This retrospective follow-up study was conducted to compare the outcomes of implant therapy at the preserved alveolar bone sites.

## Methods

This study was performed in accordance with the Helsinki guidelines and was approved by the Institutional Review Board at Seoul National University Bundang Hospital (B-2010-645-103). Patients who underwent single-tooth extraction and ARP with one of two rhBMP-2 delivery systems from October 29th, 2015 to October 6th, 2016 were chosen. Informed consent was obtained from all subjects. Among them, 28 patients whose medical records indicates implant therapy and prosthetic treatment at a preserved alveolar bone site were selected, with 14 patient in each group (Group 1: Cowell BMP; Group 2: O-BMP).

Patient demographics, treated site, characteristics of the implant fixture, implant treatment procedures, primary and secondary stability values, and amount of alveolar bone loss were collected using medical chart and radiographs (periapical view). If additional surgeries were performed at the time of implant placement, the type of and reason for surgery were noted.

The stability of each implant was measured as implant stability quotient (ISQ) according to an Osstell Mentor (Osstell AB, Gothenburg, Sweden). Primary stability was measured immediately after implant placement, and secondary stability was measured at the time of the second surgery or during the impression for the prosthesis.

**Fig. 1:**
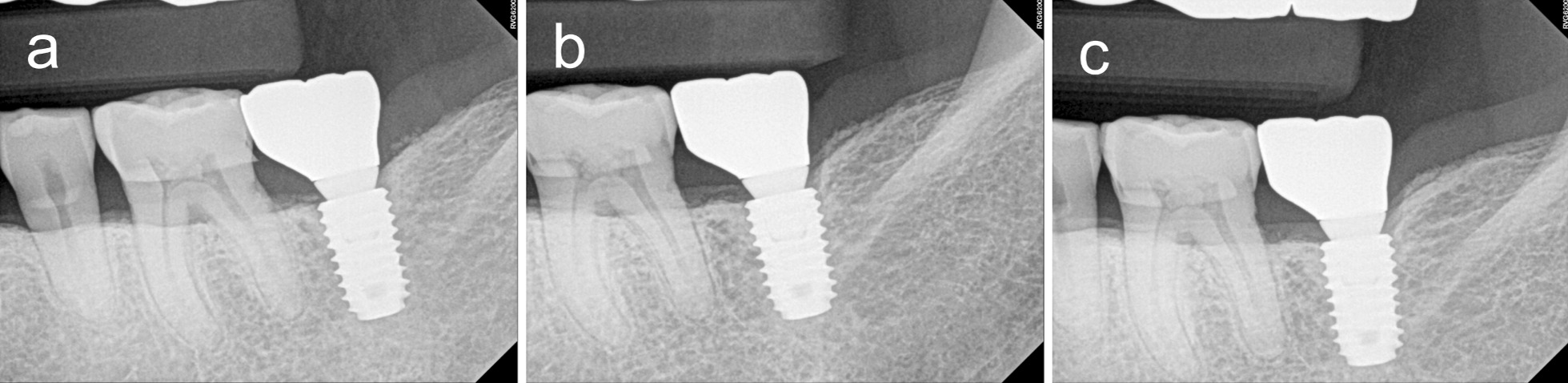
Periapical radiographs of a dental implant. **a** Immediately after loading; **b** 1 year after loading; **c** most recently

To determine the amount of marginal bone loss (MBL), radiographic analyses were performed. For measuring it, intraoral periapical radiographs produced with the paralleling technique on a digital intraoral radiographic device (Heliodent Sirona, Sirona Dental Systems Inc., NY, USA) and measurement system (PACS, INFINIT Co., Seoul, Korea) were used. MBL was defined as the mean differences of the shortest vertical distances from the mesial and distal aspect of implant shoulder to the implant-bone contact points on the aspect measured between the radiographs taken immediately after functional loading, 1 year after loading, and final observation that exceeded 1 year after loading (Fig. 1).

Implant success was evaluated with the criteria suggested by Zarb and Albrektsson in 1998. If one implant did not satisfy the criteria, the case was regarded as a failure. The time point at which the failure occurred was set as the final follow-up of that implant. Survival was defined as normal functioning of the prosthesis with no symptoms, such as mobility and patient discomfort [19].

The interval from ARP to implant placement was defined as the alveolar bone remodeling period, and that to commencement of function was defined as the total treatment period. The interval from implant placement to impression acquisition was defined as the healing period, and that to the most recent follow-up was defined as the observation period.

Differences between groups were analyzed using t-test. If the variables did not follow a normal distribution, the Mann–Whitney test was used. Statistical significance was noted a level of 95% (SPSS Ver. 25.0, SPSS Korea Institute, Inc., Seoul, Korea).

## Results

Group 1 contained 8 males and 6 females with a mean age of 59.14 ± 13.39 (38–79) years. Group 2 contained 6 males and 8 females with a mean age of 59.14 ± 10.77 (30–70) years. The surfaces of all implants were sandblasted, large grit, acid-etched (SLA). The distribution of implant types was 8 and 6 Dentium implants (Suwon, Korea), 5 and 2 Neobiotech implants (Busan, Korea), and 1 and 6 Osstem implants (Busan, Korea) in Groups 1 and 2, respectively. The mean diameter and length of the implant fixtures were 4.89 ± 0.40 (4.5−6.0) mm and 9.21 ± 1.64 (7.0−1.5) mm in Group 1, respectively, and 4.86 ± 0.50 (4.0–6.0) mm and 9.43 ± 1.16 (7.0–10.0) mm in Group 2. There were no statistical differences in diameter and length of the implants (*P* = 0.836 and 0.693, respectively) (Table 1).

Nine implants were placed in the maxilla and 5 implants were placed in the mandible in Group 1. In Group 2, 10 implants were placed in the maxilla and 4 implants were placed in the mandible. A total of 11 additional surgeries was performed during implant installation. Six sinus augmentation surgeries and 1 guided bone regeneration (GBR) procedure were completed for bone height compensation in Group 1. Of the 4 GBR procedures conducted in Group 2, half were conducted for bone height and other the half for bone width compensation (Table 2).

**Table 1:** Diameter and length of implants

		Group 1	Group 2	t	P
Diameter	(mm)	4.89 ± 0.40	4.86 ± 0.50	0.21	0.84
Length		9.21 ± 1.64	9.43 ± 1.16	− 0.4	0.69

The comparison between groups was performed with Student’s t test

**Table 2:** Diameter and length of implants and combined surgery at the time of placement

Group	No.	Site	Implant fixture (mm)		Combined surgery	
			Diameter	Length	Type	Compensation
1	1	#17	5	10	SA	Bone height
	2	#25	4.5	11.5	–	–
	3	#16	5	8.5	SA	Bone height
	4	#36	5	7	–	–
	5	#15	4.5	12	–	–
	6	#26	4.5	8.5	SA	Bone height
	7	#16	5	11.5	SA	Bone height
	8	#36	4.5	10	–	–
	9	#16	5	8.5	SA	Bone height
	10	#47	5	8	–	–
	11	#36	6	8	GBR	Bone height
	12	#36	5	7	–	–
	13	#16	5	8.5	SA	Bone height
	14	#17	4.5	10	–	–
2	1	#16	5	10	–	–
	2	#27	5	10	–	–
	3	#16	4.5	10	–	–
	4	#17	5	10	–	–
	5	#46	5	10	–	–
	6	#47	5	7	GBR	Bone height
	7	#46	6	10	GBR	Bone width
	8	#16	5	10	–	–
	9	#17	5	10	–	–
	10	#11	4	10	–	–
	11	#15	4.5	10	–	–
	12	#11	4	10	GBR	Bone width
	13	#27	5	8	–	–
	14	#46	5	7	GBR	Bone height

SA, sinus augmentation; GBR, guided bone regeneration

Primary stability (ISQ) was 69.71 ± 6.01 and 72.86 ± 9.81 for Groups 1 and 2, respectively, and secondary stability (ISQ) was 78.86 ± 5.49 and 81.64 ± 5.34, neither of which was statistically different (*P* = 0.316 and 0.185, respectively) (Table 3).

The alveolar bone remodeling period was 8.11 ± 8.92 months in Group 1 and 3.83 ± 1.13 months in Group 2 (Mann–Whitney test, *P* = 0.210). The mean total treatment period was 14.56 ± 9.03 months for Group 1 and 9.45 ± 2.56 months for Group 2 (*P* = 0.052). The mean healing period was 5.65 ± 1.66 and 4.91 ± 1.88 months for Groups 1 and 2 (*P* = 0.286), respectively. The average observation period after implant placement was 33.76 ± 14.31 months in Group 1 and 40.20 ± 9.64 months in Group 2 (Mann–Whitney test, *P* = 0.329). MBL was 0.013 ± 0.039 mm and 0.001 ± 0.022 mm for Groups 1 and 2, respectively, at 1 year after loading (*P* = 0.551), and no implant showed annual vertical bone loss greater than 0.2 mm. MBL was 0.014 ± 0.039 mm in Group 1 and 0.021 ± 0.056 mm in Group 2 at the last follow-up (*P* = 0.670) (Table 4).

**Table 3:** Primary and secondary stability of placed implants

	Group 1	Group 2	T	*P*
ISQ				
Primary	69.71 ± 6.01	72.86 ± 9.81	− 1.022	0.316
Secondary	78.86 ± 5.49	81.64 ± 5.34	− 1.361	0.185

ISQ, Implant Stability Quotient

The comparison between groups was performed with Student’s t test

**Table 4:** Marginal bone loss after 1 year of function and most recent follow ups

	Group 1	Group 2	t	*P*
MBL (mm)				
1 year of function	0.013 ± 0.039	0.001 ± 0.022	0.604	0.551
Most recent	0.014 ± 0.039	0.021 ± 0.056	− 0.431	0.670

MBL, Marginal Bone Loss

The comparison between groups was performed with Mann–Whitney test

The success rate was 100% (14/14) in Group 1 and 92.9% (13/14) in Group 2. Only one implant in Group 2 was removed, at 31.31 months after functioning due to progressive alveolar bone loss and subsequent mobility due to peri-implantitis. The survival rate was 100% (14/14) in Group 1 and 92.9% (13/14) in Group 2.

## Discussion

Many studies have reported the efficacy of horizontal and vertical bone volume preservation of ARP after tooth extraction [19–26]. This type of study would be meaningful because it shows the prognosis of implant treatment after alveolar ridge preservation and compares the long-term efficacy of two graft materials for alveolar ridge preservation.

The ISQ quantifies implant stability as a value from 1 to 100; a value less than 45 means implant failure, while a value between 60 and 70 indicates success of the implant [27]. Nedir et al. suggested that implants with an ISQ of 47 or higher should be considered stable [28]. Balleri et al. noted that successful osseointegration can be expected if the ISQ value immediately after implant placement is between 57 and 82 [29]. In the present study, the ISQ measured immediately after implant placement was 69.71 in Group 1 and 72.86 in Group 2. This suggests good-quality alveolar bone through the previous ARP, allowing excellent initial stability. At approximately 5 months after implantation, ISQ increased by 9.15 and 8.87 on average for Groups 1 and 2, respectively. Therefore, demonstrating excellent osseointegration.

The success rate of single-tooth implants varies according to placement protocol. Success rates of single-tooth implants over 1–2 years are reported as 66.7–92.0% for immediate placement after extraction and 83.3–100% for placement at 3–5 months after extraction [30–32]. In the present study, success rates of 100% in Group 1 and 92.9% in Group 2 were observed over an average of 3 years. Of all 28 implants in the two groups, 27 satisfied the success criterion, resulting in an overall success rate of 96.43%. Except for 1 implant that was removed at 31 months after functioning, 27 implants survived until the final follow-up date without mobility, progressive bone loss, or patient discomfort. This can be interpreted as a high level of positive treatment outcome in both groups.

Since there was no significant difference in groups for MBL, success rate, or survival rate, we propose that adequate-quality alveolar bone was formed with implant treatment after ARP with the two rhBMP-2 delivery systems. These results can be seen as continuation and extension of our previous study showing well-preserved extraction sockets in both groups after ARP. The change of alveolar height and width at the level of the alveolar crest at 12 weeks after ARP with Cowell BMP and O-BMP were − 0.68 ± 1.42 mm and − 0.26 ± 2.58 mm, respectively [18]. However, 6 of 14 sites in Group 1 had bone height deficiency, and 2 each of 14 sites in Group 2 showed lack of bone height and bone width, at the time of implant placement, requiring additional surgery. Despite the need for additional surgery, all of the surgeries actually performed were minimally invasive procedures such as maxillary sinus augmentation using crestal approach or GBR with a small amount of bone substitutes. Taken together, the results indicate that ARP with each of two rhBMP-2 delivery systems can reduce invasiveness in later implant surgery. In addition, previous ARP could diminish the necessity of additional subsequent bone grafting in the cases of large alveolar bone defects that could not adequately have restored the bone volume with ARP alone.

One limitation of this study is that we used implants from three companies. However, all the implants used in this study were surface-treated with SLA, bone level implant with internal connections, and possessed the same taper type. In addition, even though we used CBCT for evaluation of changes of alveolar bone in our previous study, use of periapical radiographs for this analysis in the present study is another limitation.

## Conclusions

The outcome of implant treatment after ARP with each of two different rhBMP-2 delivery systems was favorable. Therefore, we suggest that ARP using either of these systems is appropriate pre-treatment for later implant therapy.

## Data Availability

The datasets used and/or analyzed during the current study are not publicly available due to limitations of ethical approval involving the patient data and anonymity but are available from the corresponding author on reasonable request.
